# Structural Discrimination of Networks by Using Distance, Degree and Eigenvalue-Based Measures

**DOI:** 10.1371/journal.pone.0038564

**Published:** 2012-07-06

**Authors:** Matthias Dehmer, Martin Grabner, Boris Furtula

**Affiliations:** 1 UMIT, Institute for Bioinformatics and Translational Research, Hall in Tyrol, Austria; 2 University of Kragujevac, Faculty of Science, Kragujevac, Serbia; Universidad de Zarazoga, Spain

## Abstract

In chemistry and computational biology, structural graph descriptors have been proven essential for characterizing the structure of chemical and biological networks. It has also been demonstrated that they are useful to derive empirical models for structure-oriented drug design. However, from a more general (complex network-oriented) point of view, investigating mathematical properties of structural descriptors, such as their uniqueness and structural interpretation, is also important for an in-depth understanding of the underlying methods. In this paper, we emphasize the evaluation of the uniqueness of distance, degree and eigenvalue-based measures. Among these are measures that have been recently investigated extensively. We report numerical results using chemical and exhaustively generated graphs and also investigate correlations between the measures.

## Introduction

Structural analysis of graphs has been an outstanding problem in graph theory for several decades [1]–[4]. A challenging problem in this theory is to investigate structural features of the graphs and their characterization. Another important task is to quantify the structural features of graphs, as well as their complexity [2], [3], [5], [6]. The former relates to developing measures such as the clustering coefficient or the average distance of a graph [7]. The latter relates to deriving complexity indices for graphs, which are often called structural descriptors/measures or topological indices [8]–[11].

In this paper, we deal with evaluating the uniqueness, discrimination power or degeneracy of special graph measures for investigating graphs holistically (in contrast to local graph measures) [12]. A descriptor is called degenerate if it possesses the same value for more than one graph. In view of the large body of literature on structural graph measures [2], [3], [5], [13], the degeneracy problem has been somewhat overlooked in graph theory. In fact, the uniqueness of structural descriptors has been investigated in mathematical chemistry and related disciplines for discriminating the structure of isomeric structures and other chemical networks [14]–[16]. A detailed survey on the uniqueness of topological indices by using isomers and hexagonal graphs has been given by Konstantinova [16]. For more related work, see also [17].

To date, no complete graph invariant, i.e., a measure that is fully unique on general graphs, has been found. Indeed, some measures turned out to be complete by using special sets of graphs [15], [17], [18]. In a more general context, i.e., by using graphs without structural constraints, any topological graph measure has a certain kind of degeneracy, which also depends on the mathematical method to define the measure, see [19], [20]. A highly discriminating graph measure is desirable for analyzing graphs; hence, measuring the degree of its degeneracy is important for understanding its properties, limits and quality.

The main contribution of this paper is to investigate to what extent known degree, distance and eigenvalue-based measures are degenerate. Among the measures we examine (see Table 1) are the recently developed geometric-arithmetic indices [21], [22], the atom-bond connectivity index [23] and the Estrada index [24], which is based on the eigenvalues of a special graph-theoretical matrix [25], here the adjacency and Laplacian matrix. It turns out that some of the measures based on distances and eigenvalues are highly unique in exhaustively generated graphs (e.g., see Table 2). Using these graphs is a greater challenge than only using isomeric structures, as exhaustively generated graphs do not possess any structural constraints. However, it is clear that other distance or eigenvalue-based measures exist that possess only low discrimination power [26], implying that the uniqueness of a measure crucially depends on its mathematical composition and the graph class under consideration.

**Table 1:** The topological indices used for determining the value distributions and correlation plots.

Index Name	Symbol
Atom-bond connectivity index [23]	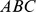
Augmented Zagreb index [40]	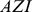
Variable Zagreb index [41]	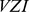
Modified Zagreb index [42]	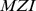
Narumi-Katayama index [43]	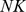
Distance degree centric index [8], [44]	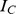
Offdiagonal complexity [45]	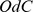
Medium articulation [46]	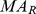
Degree-degree association index [29]	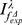
First geometric-arithmetic index [21]	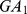
Second geometric–arithmetic index [22]	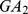
Third geometric–arithmetic index [31]	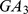
Efficiency complexity [26]	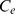
Graph energy [47]	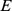
Laplacian energy [48]	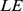
Estrada index [24]	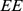
Laplacian Estrada index [49]	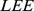
Spectral radius [10]	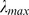
Graph index complexity [26]	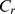
Balaban index [19]	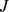
Degree information index [8]	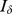
Topological information content [6]	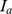
Vertex complexity [50]	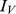

## Methods and Results

### Uniqueness of Topological Descriptors

In this section, we present numerical results when evaluating the uniqueness of certain topological descriptors. Note that a summary of the topological indices used in this paper can be found in Table 1. As mentioned, the discrimination power of these measures has not yet been evaluated extensively on a large scale. Therefore, the results might be useful for gaining deeper insights into these measures and for enabling implications when designing novel topological descriptors. As usual, we use the measure
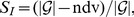
(1)which was called the sensitivity by Konstantinova [15], for evaluating the uniqueness of an index 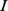. Clearly, 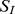 depends on a graph class 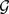; ndv are the values that cannot be distinguished by 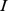, and 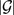 is the size of the graph set. Now, we start interpreting the results by considering Table 2 and observe that we have arranged the used descriptors into four groups. We also emphasize that the values in Table 2 have been calculated by using the graph classes 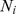, 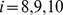. These are the classes of exhaustively generated non-isomorphic, unweighted and connected graphs with 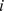 vertices each. The cardinalities 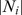 are also depicted in Table 2.

**Table 2:** Exhaustively generated sets of non-isomorphic and generated graphs.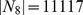, 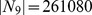 and 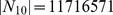.

	*N*_8_		*N*_9_		*N*_10_	
Index	ndv	*S*	ndv	*S*	ndv	*S*
**Degree-based Measures**						
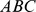	8520	0,233606	241793	0,073874	11539714	0,015095
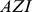	8520	0,233606	241777	0,073935	11539377	0,015123
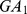	8522	0,233426	242009	0,073047	11542066	0,014894
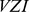	10500	0,055501	258286	0,010702	11704386	0,001040
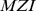	10496	0,055860	258293	0,010675	11704428	0,001036
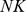	10974	0,012863	260925	0,000594	11716377	0,000017
**Information-theoretic Measures**						
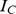	11116	0,000090	261079	0,000004	11716570	0,000000
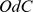	10731	0,034722	259967	0,004263	11713337	0,000276
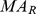	10879	0,021409	260576	0,001930	11715462	0,000095
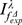	385	0,965368	6016	0,976957	609204	0,948005
**Distance-based Measures**						
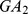	1044	0,906090	40014	0,846737	3693236	0,684785
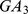	663	0,940362	15228	0,941673	673972	0,942477
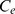	11076	0,003688	261020	0,000230	11716455	0,000010
**Eigenvalue-based Measures**						
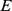	1628	0,853558	47577	0,817769	2413055	0,794048
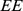	751	0,932446	26457	0,898663	1460054	0,875386
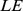	5098	0,541423	59542	0,771940	2338347	0,800424
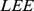	1013	0,908878	23393	0,910399	718156	0,938706
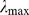	2003	0,819825	48120	0,815689	2137087	0,817601
**Non-information-theoretic Measures**						
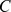	10950	0,015022	260861	0,000839	11716146	0,000036
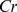	1779	0,839975	44652	0,828972	2098604	0,820886

For the degree-based indices, it is not surprising that these measures have only little discrimination power, as many graphs can be realized by identical degree sequences. This effect is even stronger if the cardinality of the underlying graph set increases, see Table 2. The highest discrimination power among the indices of this class has the 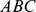 index. This is in accordance with the well-known fact that the degeneracy of topological descriptors decreases in the following order: 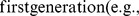
NK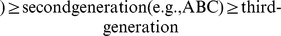, see [27]. Recall that first-generation indices are integer measures derived from integer local vertex invariants such as vertex degrees or distances sums [28]. Second-generation indices are real numbers derived from integer local vertex invariants [28]. Third-generation indices are real numbers derived from real local vertex invariants [28].

Most of the information-theoretic measures (e.g., 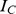, 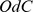) we have evaluated in this study are based on grouping elements (e.g., vertices, degrees, etc.) in equivalence classes [6], [8] to determine probability values. We observe that the uniqueness of these measures is also low. In contrast, the degree-degree association index 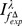 [29] is highly discriminating for all three graph classes [30]. Surely, a reason for this is the fact that this measure is non-partition-based, as probability values have been assigned to each vertex in the graph by using the special information functional 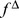, see [29]. Note that 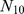 contains almost 12 million graphs. Calculating the discrimination power of the distance-based measures, such as the second or third geometric-arithmetic indices [22], [31], leads to a somewhat surprising result: the uniqueness for 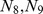 and 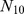 is very high, but recall that they belong to the class of so-called second-generation indices [27]. Again, we see that the composition of the graph invariant (here, distances) to define the measure is crucial.

If we compare the sensitivity values (using Equation 1) of some second-generation indices, e.g., the geometric-arithmetic indices with some of the third-generation indices (information-theoretic and eigenvalue-based measures), we observe that the uniqueness of e.g., 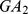, 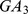 is unexpectedly high. In particular, the high uniqueness of 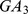 for graphs 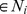, 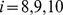, is probably caused by the fact that its calculation is based on distances between edges. As the number of edges lies in the interval 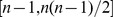, the range of the third geometric-arithmetic index is 0 to 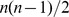 [32], and the probability that two graphs have different index values is certainly larger than in the case when the number of edges would be fixed. This hypothesis can be supported by comparing the values of the sensitivity index (using Equation 1) of the 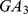 index shown in Tables 2 and 4. Thus, the sensitivity index resulting from 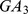 shown in Table 2 is greater than 0.94 (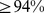), while, if the number of edges is fixed, see Table 4, the corresponding sensitivity index is less than 0.02 (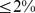). Using this idea again, it can be understood why the sensitivity index of 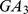 (see Table 2) does not decrease with the number of vertices.

Let us turn to the uniqueness of some eigenvalue-based measures such as the graph energy 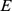, the Estrada index 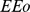 and the Laplacian Estrada index 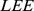. As expected, it is high because these measures belong to the class of third-generation indices (e.g., information-theoretic measures). We point out that the sensitivity index of the graph energy 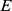 and Laplacian energy 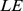 could be affected by rounding errors. The reason for this is based on the fact that the difference between the values of 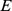 and 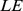 for some graphs is less than 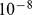 [33]. However, since the number of such graphs is very small, see [33], this does not strongly affect the computation of the uniqueness of 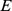 and 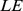 measured by 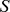 and ndv. In particular, the Estrada and Laplacian Estrada indices possess high uniqueness for all three graph classes 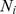. To give some arguments for this, recall their definitions, namely
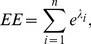
(2)

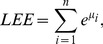
(3)where 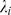 and 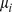 are the eigenvalues of the adjacency and Laplacian matrices, respectively. Knowing that 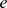 is irrational and transcendental, it can be presumed that any power and the sum thereof is also irrational and transcendental. Hence, the graphs with the same Estrada (Laplacian Estrada) index are isospectral.

In addition, the uniqueness of these measures is quite stable, and the same holds for 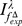. This means that there is only very little dependency between their uniqueness and the cardinality of the underlying graph set. Clearly, this result demonstrates that certain measures/functions based on the eigenvalues of graphs possess a high discrimination power. This contradicts the widely assumed hypothesis that graph spectra are not feasible to discriminate graphs properly because of the existence of isospectral graphs, see [34], [35]. Another positive example can be found in [36] where Dehmer et al. presented spectrum-based measures based on a probability distribution of structural values with low degeneracy.

In Table 3 and Table 4, we have also evaluated the discrimination power of the measures using isomers and chemical trees. In particular, we use the isomeric classes 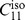 and 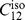 containing all isomers with 11 and 12 vertices, see Table 3. The numerical results are quite similar to Table 2. However, when evaluating the indices by using the classes of chemical trees 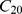, 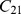 and 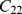, we see that the discrimination power of 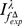 deteriorates significantly. To better understand this, note that the information functional 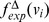 relies on determining the shortest paths for all 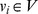 and, then, degree-degree associations thereof resulting in 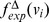, see [29]. Finally, when applying this measure to trees, the reason for the deterioration of its uniqueness could be understood by the occurrence of a large number of paths possessing similar length and, hence, resulting in very similar probability values and entropies. Interestingly, the eigenvalue-based measures 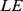 and 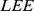 possess high uniqueness, and whose values are almost independent of the cardinality of the graph sets. Thus, these measures turned out to be quite feasible to discriminate chemical trees uniquely.

**Table 3:** Chemical isomers with 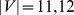.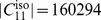, 
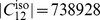
.

	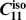		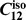	
Index	ndv	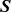	ndv	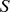
**Degree-based Measures**				
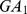	160063	0,001441	738685	0,000329
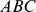	160089	0,001279	738714	0,000290
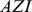	160093	0,001254	738721	0,000280
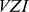	160290	0,000025	738924	0,000005
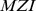	160290	0,000025	738924	0,000005
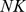	160293	0,000006	738927	0,000001
**Information-theoretic Measures**				
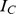	160292	0,000012	738925	0,000004
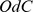	160281	0,000081	738916	0,000016
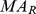	160291	0,000019	738926	0,000003
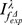	1479	0,990773	18852	0,974487
**Distance-based Measures**				
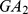	23548	0,853095	118000	0,840309
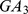	11046	0,931089	60597	0,917993
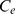	160036	0,001610	738454	0,000641
**Eigenvalue-based Measures**				
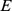	24417	0,847674	110075	0,851034
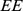	19590	0,877787	88842	0,879769
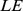	22982	0,856626	104151	0,859051
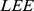	10062	0,937228	39634	0,946363
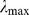	28195	0,824104	117781	0,840606
**Non-information-theoretic Measures**				
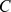	160293	0,000006	738927	0,000001
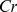	21432	0,866296	91321	0,876414

**Table 4:** Chemical trees with 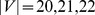.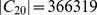, 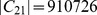, 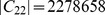.

	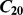		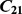		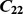	
Index	ndv	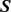	ndv	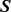	ndv	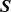
**Degree-based Measures**						
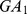	366257	0,000169	910662	0,000070	2278593	0,000029
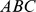	366303	0,000044	910710	0,000018	2278640	0,000008
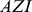	366303	0,000044	910710	0,000018	2278640	0,000008
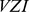	366318	0,000003	910722	0,000004	2278657	0,000000
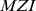	366318	0,000003	910722	0,000004	2278657	0,000000
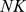	366318	0,000003	910725	0,000001	2278657	0,000000
**Information-theoretic Measures**						
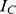	366283	0,000098	910688	0,000042	2278608	0,000022
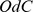	366311	0,000022	910718	0,000009	2278652	0,000003
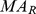	366317	0,000005	910725	0,000001	2278657	0,000000
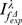	196124	0,464609	544432	0,402200	39396	0,982711
**Distance-based Measures**						
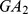	362628	0,010076	904971	0,006319	2266566	0,005307
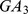	362171	0,011323	904971	0,006319	2270582	0,003544
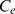	319073	0,128975	813531	0,106723	2081010	0,086739
**Eigenvalue-based Measures**						
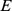	93204	0,745566	228831	0,748738	479746	0,789461
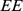	87656	0,760711	224579	0,753407	525472	0,769394
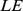	544	0,998515	880	0,999034	1275	0,999440
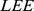	292	0,999203	509	0,999441	842	0,999630
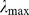	130783	0,642981	318330	0,650466	675147	0,703708
**Non-information-theoretic Measures**						
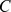	366318	0,000003	910725	0,000001	2278657	0,000000
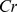	69592	0,810024	160051	0,824260	316572	0,861071

### Value Distributions

**Figure 1:**
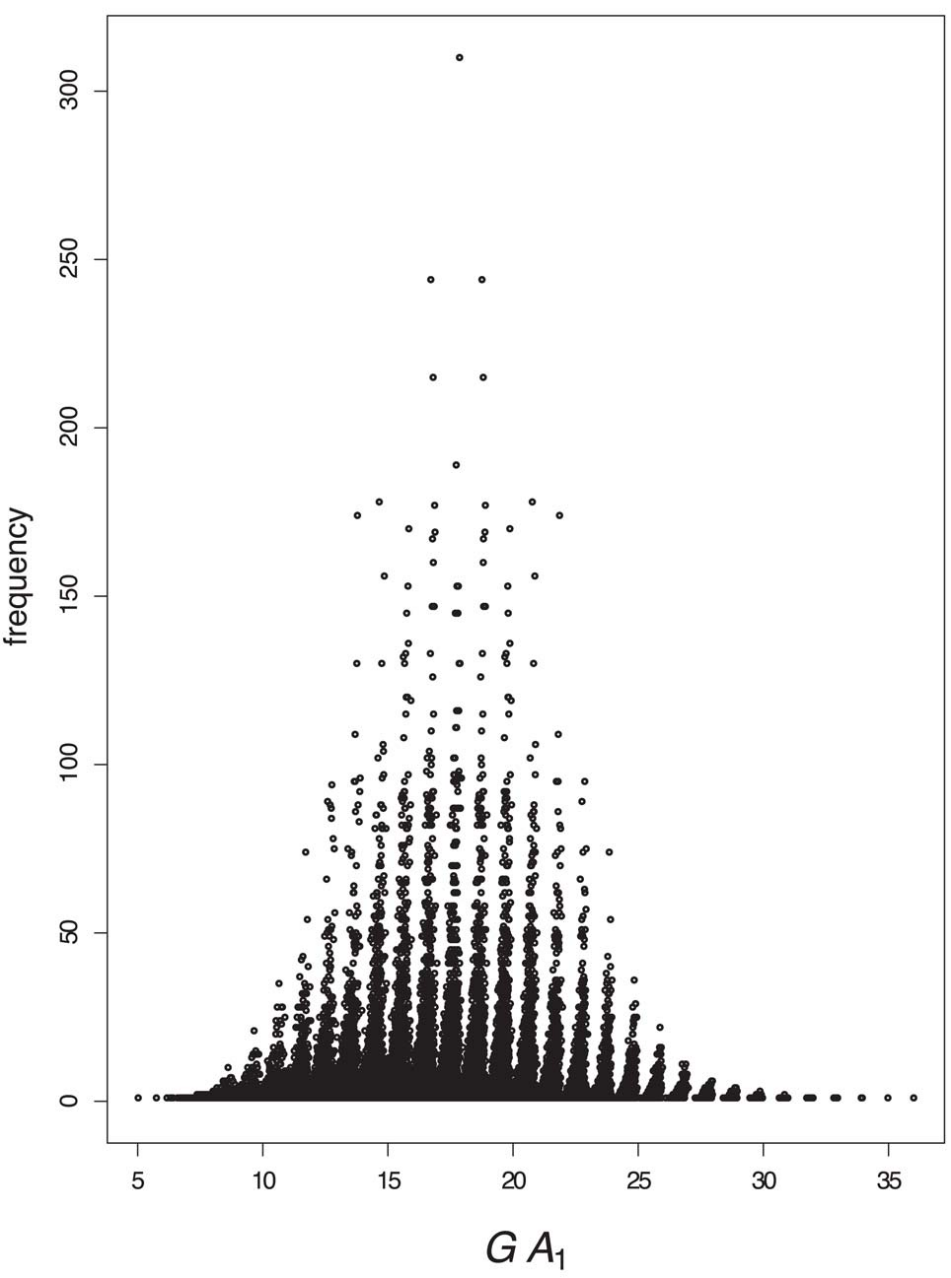
Value distribution for* GA_1_*.

**Figure 2:**
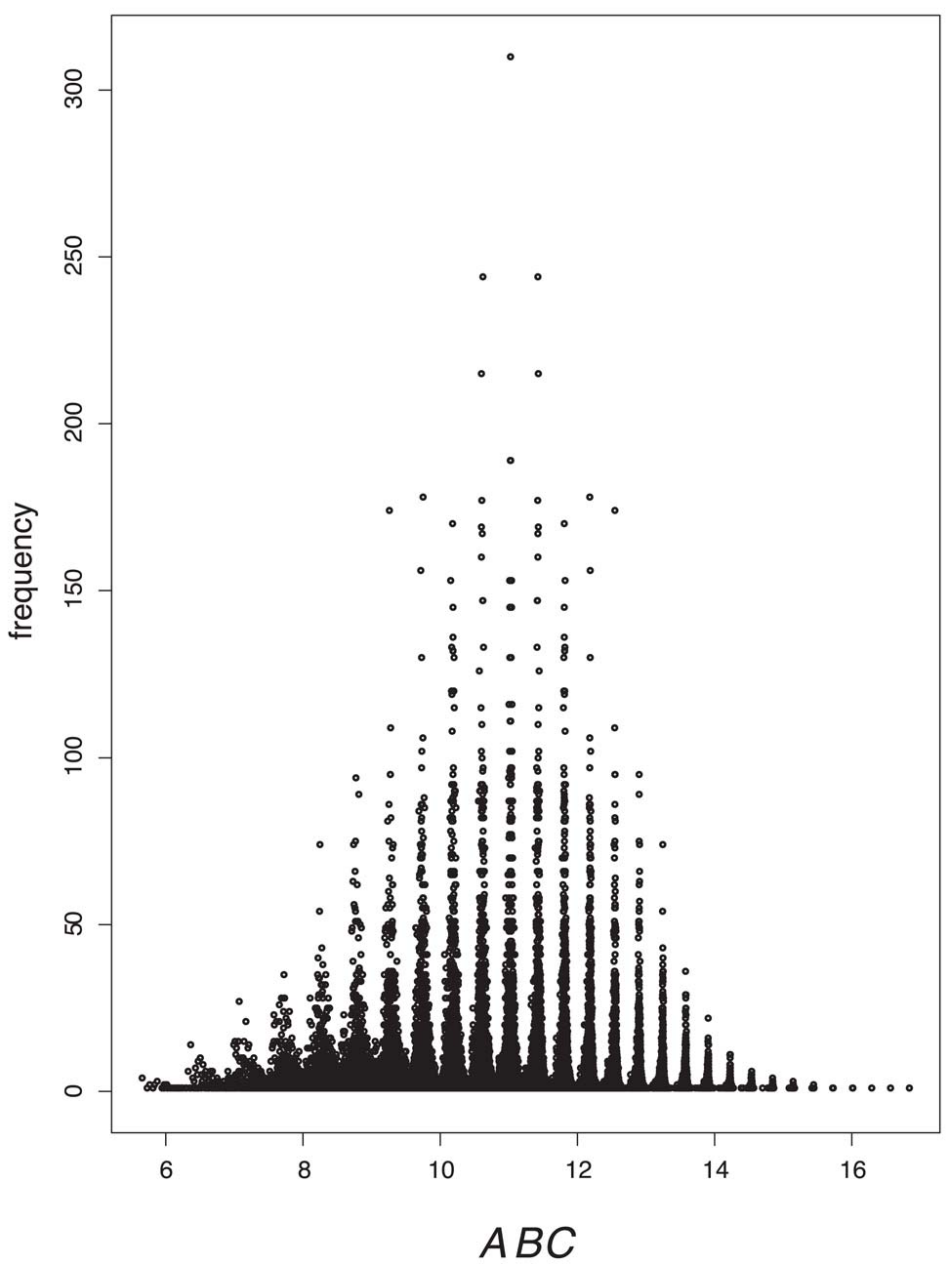
Value distribution for *ABC.*

In order to tackle the question of what kind of degeneracy the measures possess, we plot their characteristic value distributions. The 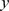-axis is the absolute frequency of the graphs, with a certain index value depicted on the 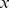-axis. For a graph class, we use the class of exhaustively generated non-isomorphic, connected and unweighted graphs denoted by 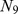. We start with Figures 1 and 2 and observe the vertical strips, indicating that a large number of graphs have quite similar index values discretely distributed on a certain interval. In addition, the hull of these value distributions looks like a Gaussian curve. This means that by using 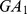 and 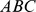, there exist many degenerate graphs possessing quite similar index values where the hull of the distributions forms a Gaussian curve.

**Figure 3:**
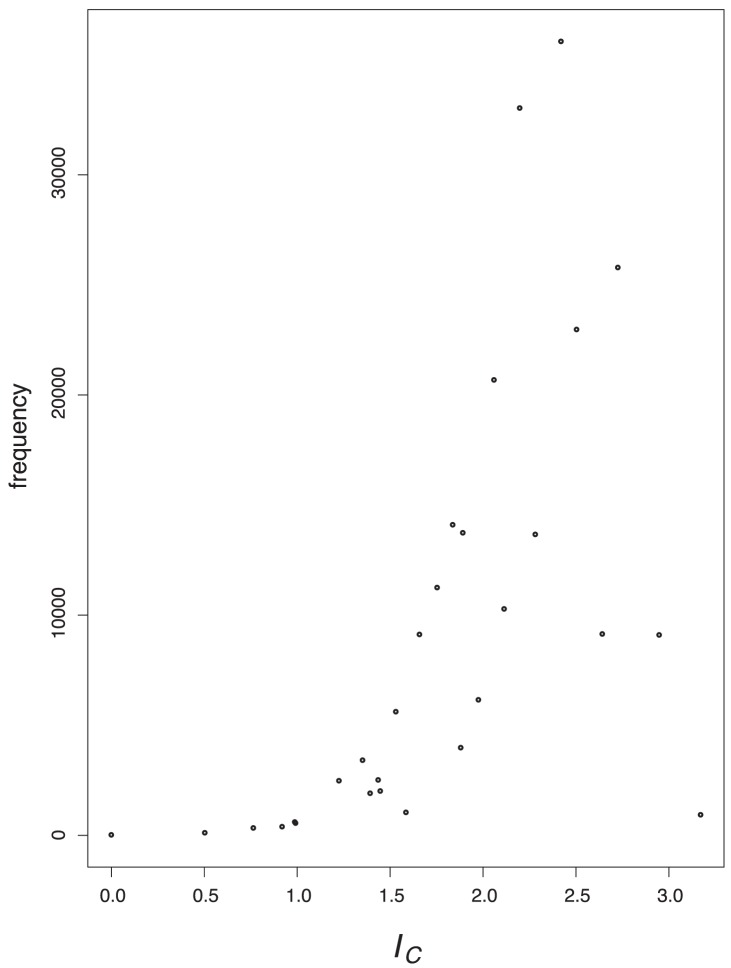
Value distribution for *I_C_.*

**Figure 4:**
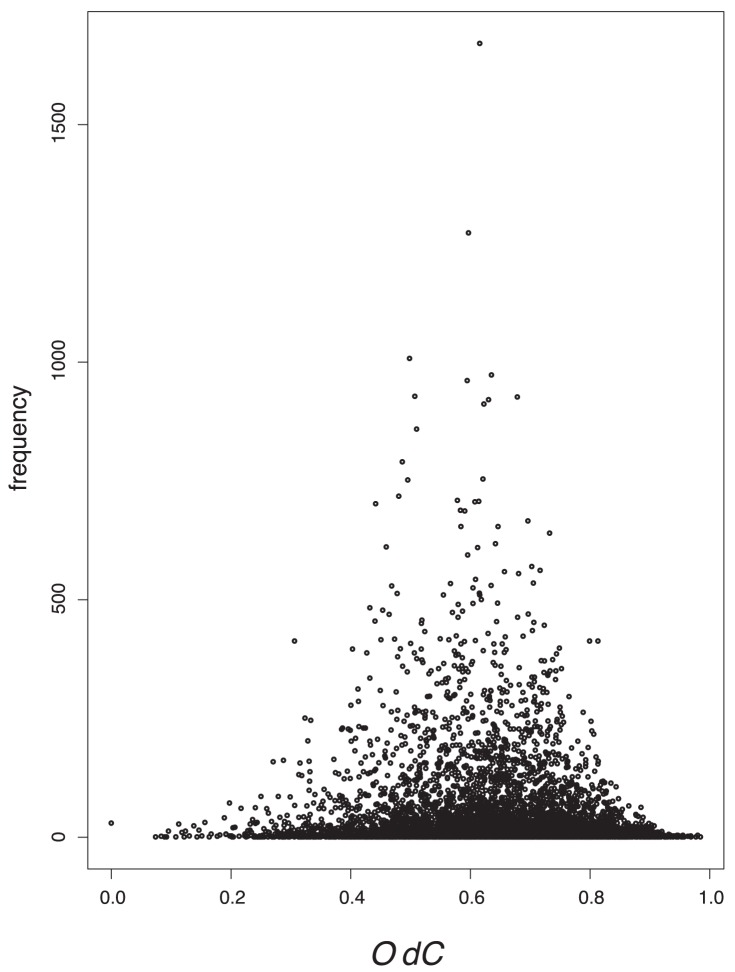
Value distribution for *OdC.*

**Figure 5:**
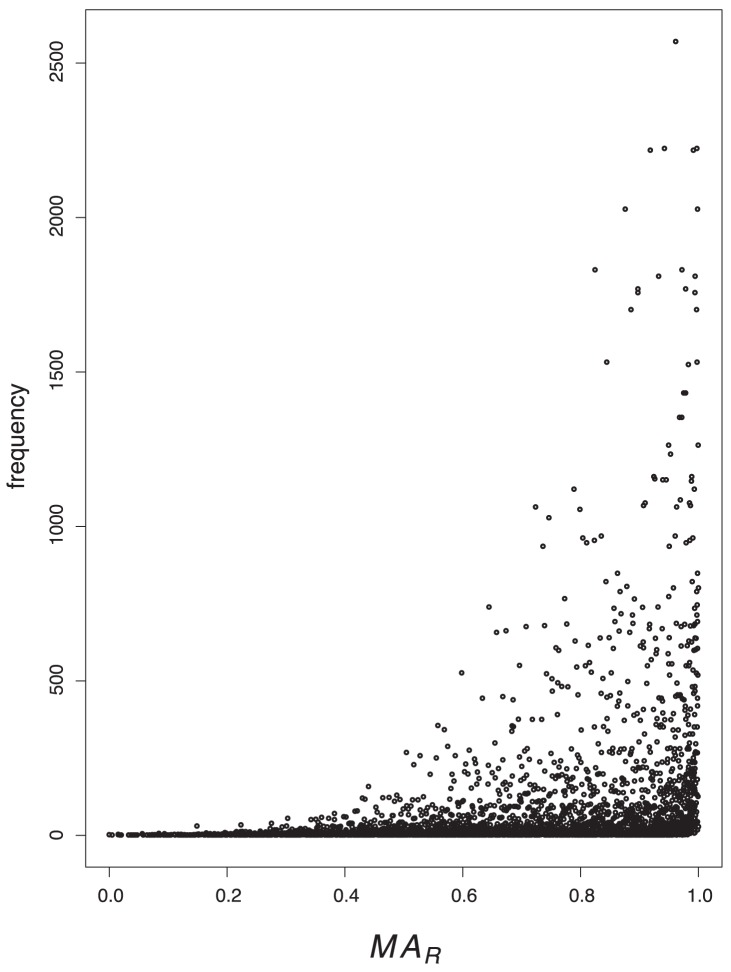
Value distribution for *MA.*

**Figure 6:**
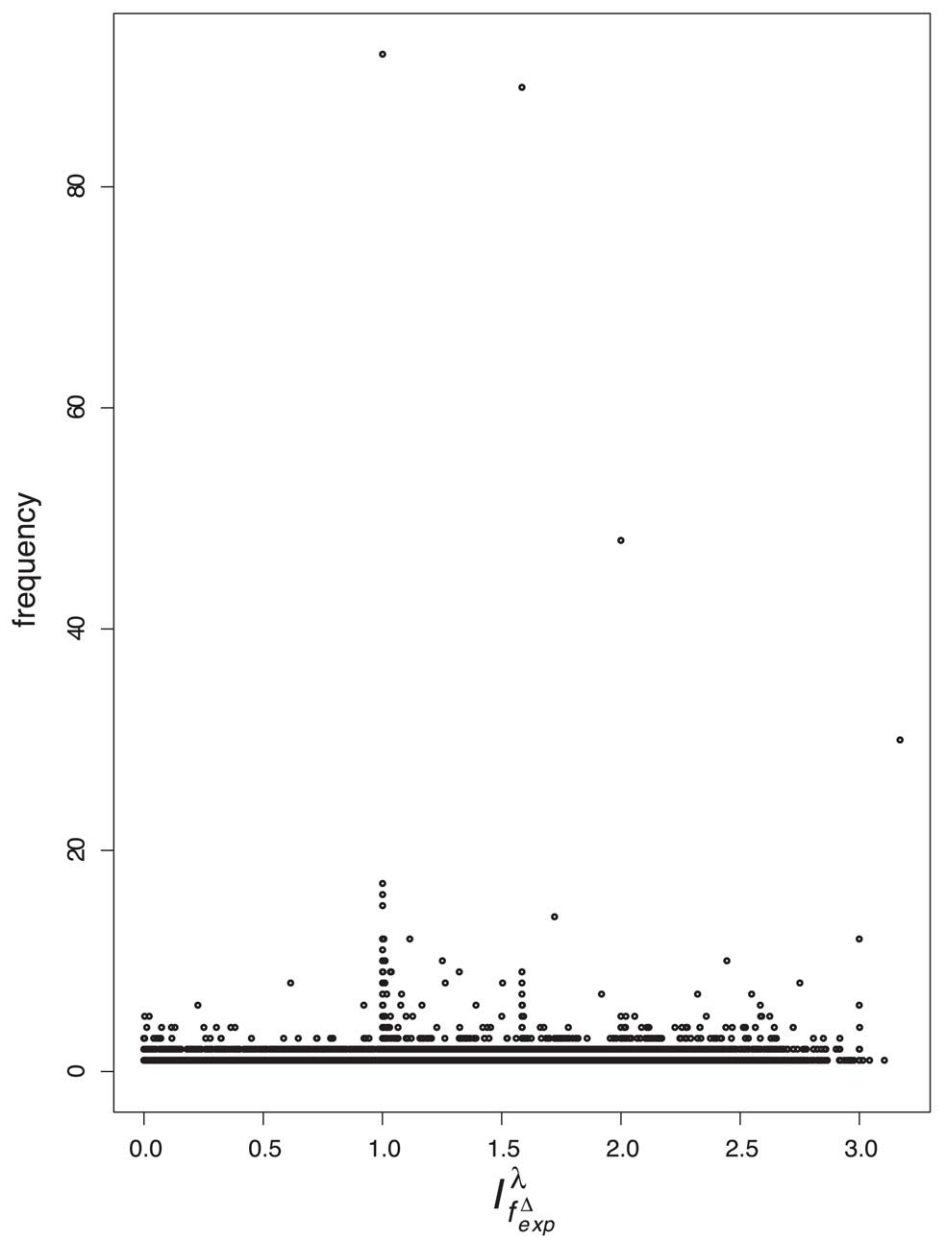
Value distribution for 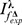.

As we can see from Figures **3**, **4**, **5**, **6**, the value distribution (and in fact the distribution of degenerate graphs) when considering the information-theoretic measures is significantly different. We start with 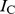, and see that the value distribution is quite scattered, i.e., there are no regions in which the graphs are closely clustered. In contrast, the values of 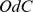 are rather clustered. Similarly, this also holds for 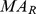 and observe that all three measures (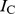, 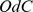 and 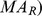 are highly degenerate on 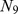. But, the degree-degree association index 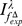 possesses a high discrimination power (see Figure 6). In particular, we see that there exist only a very few degenerate graphs whose index values exploit the entire domain.

**Figure 7:**
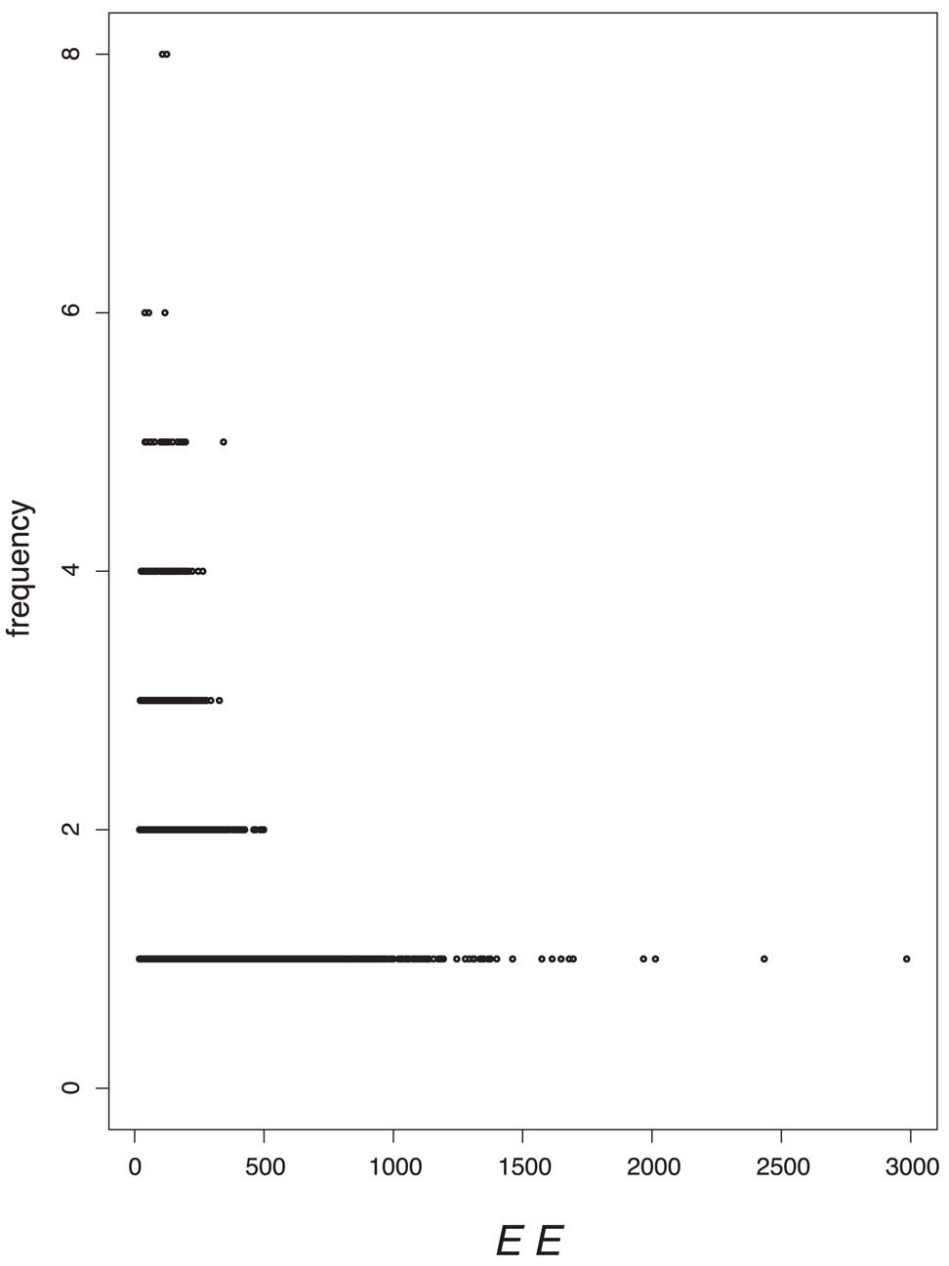
Value distribution for *E.*

**Figure 8:**
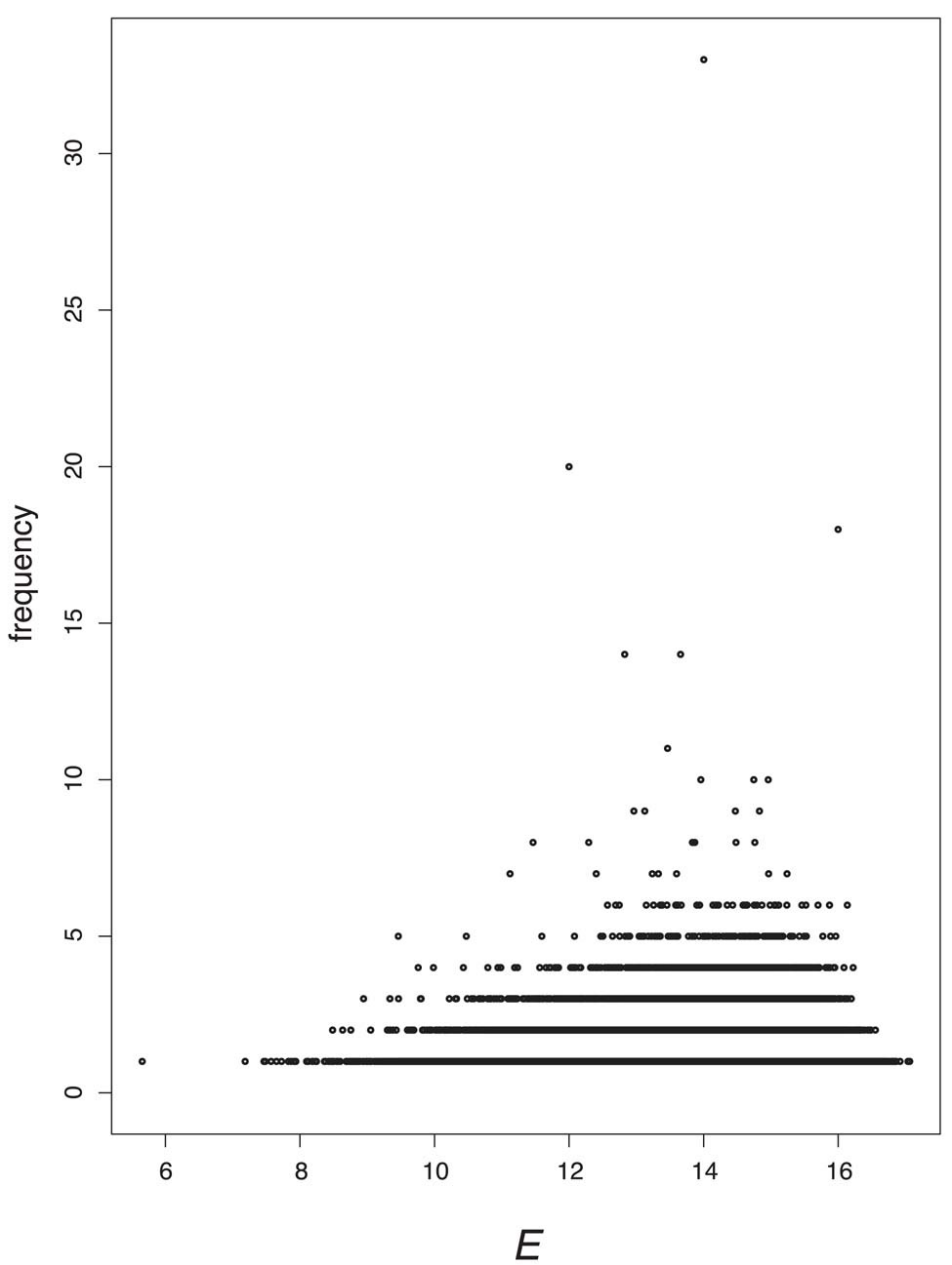
Value distribution for *EE.*

The results of plotting the value distributions for the eigenvalue-based measures graph energy 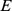 and Estrada index 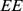 are depicted in Figures 7 and 8. We see that they possess a high discrimination power and observe the horizontal strips. This means that a certain number of graphs (e.g., 2, 4, etc.) possess index values in a certain domain. When considering Figure 7, the horizontal strip for 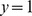 indicates the low degeneracy of this measure. This is similar for the 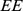 shown in Figure 8.

### Correlations Between Indices

**Figure 9:**
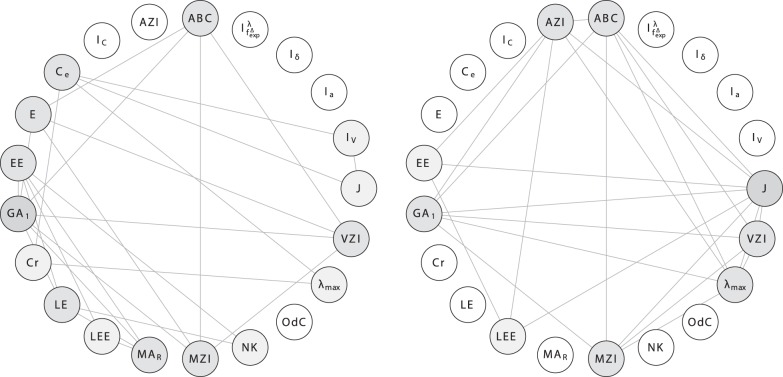
Left: Correlation network 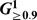 inferred from 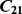. Right: Correlation network 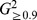 inferred from 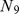 **.**

**Figure 10:**
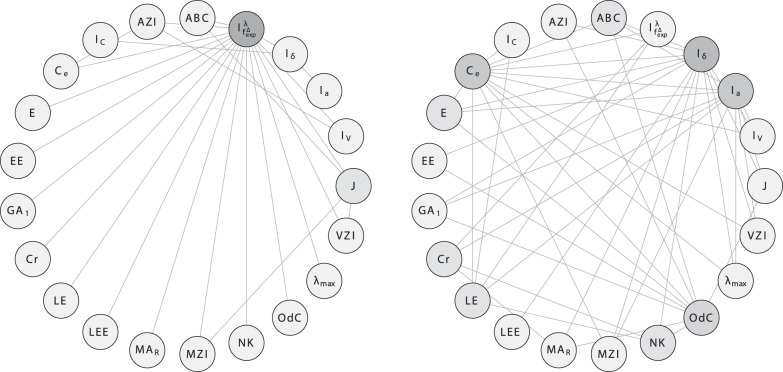
Left: Correlation network 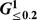 inferred from 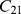. Right: Correlation network 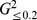 inferred from 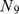 **.**

**Figure 11:**
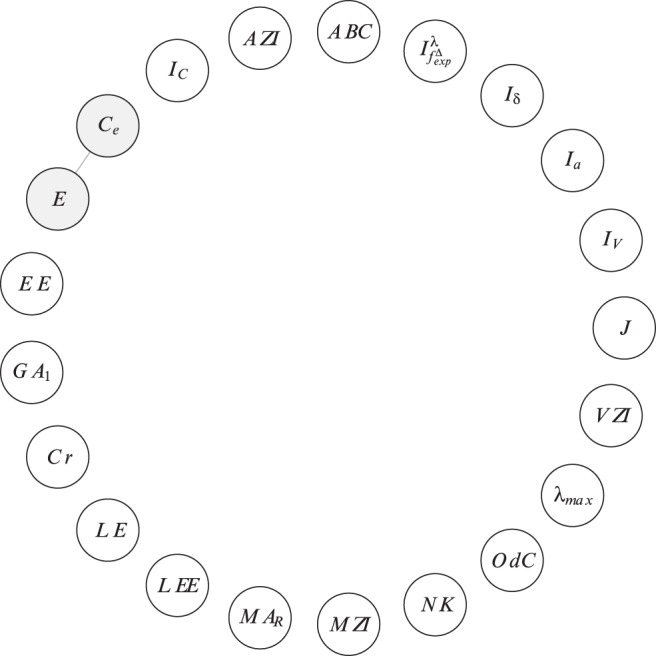
Correlation network 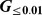 inferred from 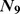.

**Figure 12:**
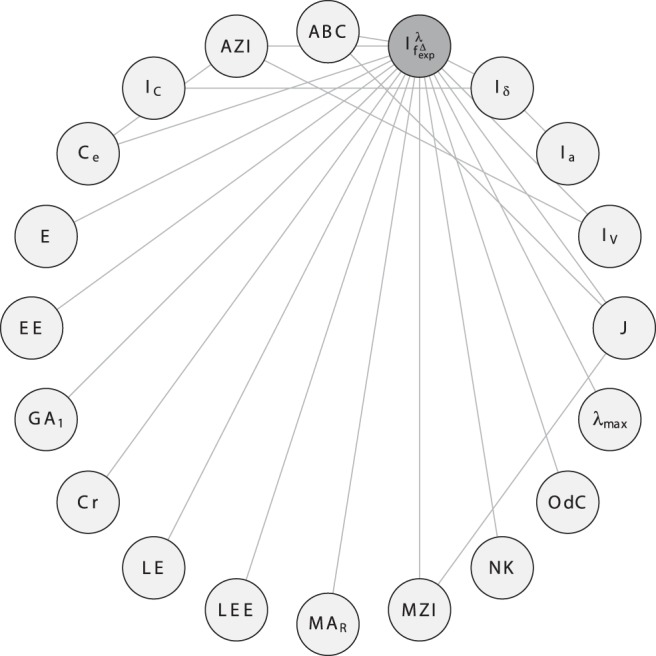
Correlation network 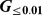 inferred from 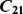.

**Figure 13:**
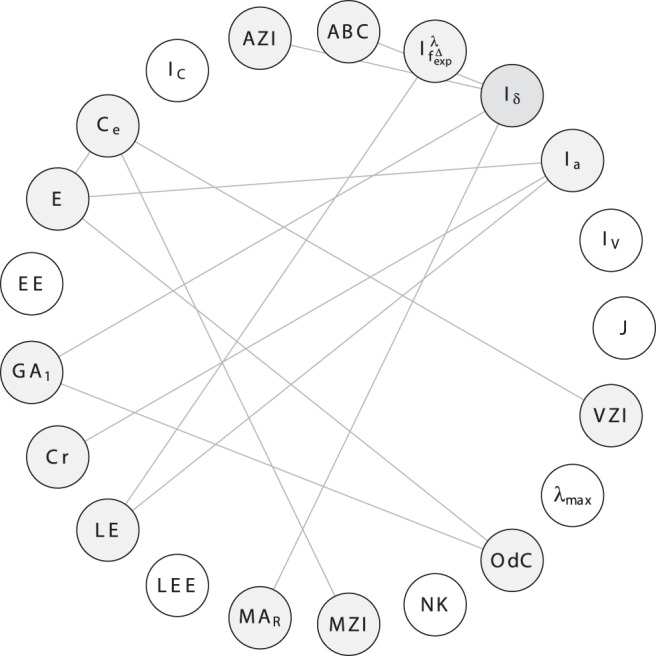
Correlation network 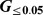 inferred from 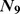.

**Figure 14:**
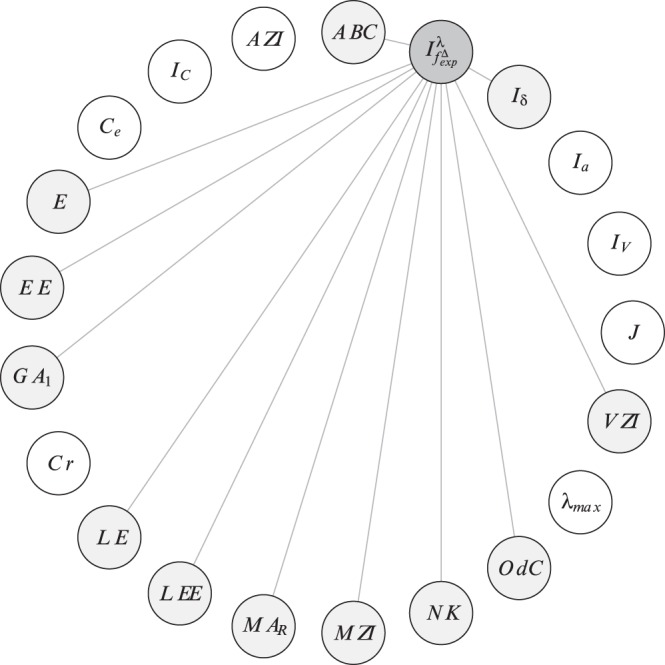
Correlation network 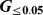 inferred from 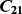.

In order to investigate the correlation ability of the topological indices, we calculate the linear correlation between them and depict the results as correlation networks. More precisely, the linear correlation between the descriptor values of two data vectors has been computed according to the method of Pearson [37]. In the depicted plots of the correlation networks, the calculated Pearson Product-Moments have then been used as edge weights for labeling the edges connecting the vertices representing the compared descriptor pairs. The correlation networks are shown in Figures **9**, **10**, **11**, **12**, **13**, **14**.

We use the graph classes 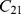 and 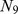, and choose different thresholds for the correlation coefficient, resulting in different networks.

#### Definition 1

Let 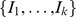 be a set of topological indices defined on a graph class 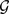 and let 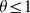. The vertex and edge set of the correlation network 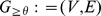 inferred from 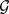 is defined by
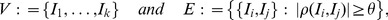
(4)where 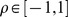 is the correlation coefficient.

#### Definition 2

Let 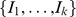 be a set of topological indices defined on a graph class 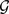 and let 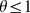. The vertex and edge set of the correlation network 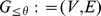 inferred from 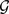 is defined by
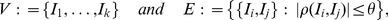
(5)where 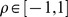 is the correlation coefficient.

We start interpreting the results by considering the left-hand side of Figure 9. The vertices of the graph 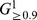 represent indices that are highly correlated (here, 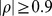) by using the graph class 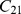. In all correlation graphs, hub vertices, i.e., those with a high degree, are colored in gray. In particular, the grayer the color of a vertex is, the higher its degree.

In 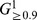, the first geometric-arithmetic index (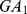) and other measures are highly correlated with other indices that belong to different groups, e.g., degree-based and eigenvalue-based, etc. In addition, graph energy (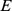) and Estrada index (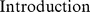) are highly correlated with other measures such as the Modified Zagreb index (degree-based). By using the graph class 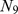, we obtain the same type of correlation network denoted by 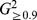. Observe that the connectedness of this network is similarly high in 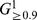, however, there exist new hubs. For instance, the Balaban 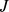 and the augmented Zagreb index (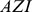) index represent such vertices, i.e., they are highly correlated with other indices from different paradigms such as degree-based and eigenvalue-based measures. Interestingly, the uniqueness (measured by ndv and 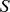) of, e.g., 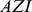 and 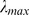 by using 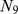 is higher than by taking 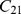 into account. Nevertheless, these indices (and others) possess larger neighborhoods compared to 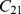. This means that they contain more highly correlated vertices adjacent to 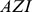 and 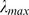 than by using 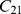. One would have expected this in a reverse order as the isomers (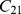) are structurally more similar among each other than the graphs contained in 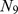. It is likely that the reasons for this are different structural characteristics captured by the underlying graphs of 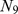 and 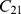.

For studying indices that are only slightly correlated, firstly consider 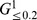 in Figure 10. We see that the degree-degree association index (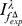) is a hub vertex, i.e., there is only a small correlation. That means 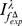 (by using 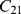) captures structural information significantly different compared to almost all other measures (representing vertices) in this network. If we consider 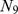 as a graph set, we observe that 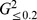 has more hubs than 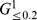. For instance, 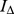 and 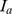 represent hubs and therefore possess only a small correlation with other measures from different paradigms. This also implies that the structural characteristics of the graphs 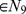 are different to those 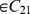. Also, the hubs in 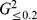 could serve as potential candidates to be tested for solving QSAR/QSPR problems [38] as they capture structural characteristics differently (compared to classical indices) and some (e.g., efficiency complexity and offdiagonal complexity) have not yet been used in mathematical chemistry and drug design. In addition, it would be interesting to examine their ability for classifying graphs optimally by using supervised learning techniques, e.g., see [39].

To finalize this section, we consider Figures 11, 12, 13, 14. We have also plotted the evolution of the correlation networks for 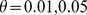, and have obtained the networks 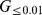 and 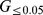 for both 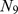 and 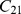, respectively. From Figure 11, we see that by using 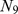, the measures 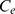 and 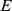 are highly uncorrelated (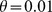). In addition, the degree-degree association index 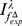 and 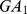 are highly uncorrelated by using 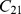 (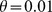). If we now choose 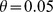 for 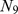 and 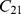, the resulting networks (see Figures 13 and 14) also show highly uncorrelated indices. Starting with 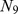 (see Figure 13), far more indices are highly uncorrelated (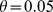) compared with Figure 11. These indices belong to different paradigms (degree-based, information-theoretic, etc.). But when considering the graph class 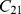 (see Figure 14), only the degree-degree association index 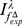 is highly uncorrelated (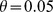) with many other indices. It is clear that the differences between these correlation networks are clearly induced by the structural differences (factors such as cyclicity and connectedness, which contribute to the complexity of the graphs) of the graph classes. Note that we obtained a similar result by comparing 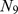 and 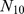 (instead of 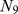 and 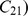. Figure 14 expresses that by using trees, 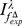 captures structural information significantly different than many other non-information-theoretic indices such as 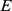, 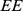, etc. We hypothesize that this result also holds for other tree classes as well. As mentioned above, the index 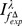 could be used to characterize graphs for problems in structural chemistry or QSAR, with the aim that it solves a particular problem (e.g., QSAR/QSPR) better than existing indices which have already been used.

### Summary and Conclusion

In this paper, we have explored to what extent degree and eigenvalue-based measures are degenerate. To tackle this problem, we used exhaustively generated undirected, connected and non-isomorphic graphs and chemical graphs. Interestingly, we found that some recently developed distance-based measures, e.g., 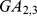, have a much better uniqueness than measures that are known to be highly unique for chemical graphs, e.g., the Balaban 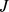 index. Note that the results for the Balaban 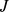 index by using the classes 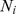, 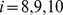, have been reported in an earlier paper [30]. Equally, some of the eigenvalue-based measures such as 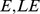 and 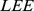 possess high discrimination power for all graph classes that we examined in this paper. This shows that such measures for discriminating graphs structurally can be feasible, despite the existence of isospectral graphs. A strong point of all measures (except the topological information content for large graphs, as it relies on determining their automorphism groups) used in this study is their polynomial time complexity. Hence, they could also be applied to large complex networks. First studies of examining the uniqueness of structural measures by using gene networks inferred from high-throughput data are under development. We will also examine the relationship between the uniqueness of a measure and the ability to classify graphs meaningfully.
